# Evaluation of the SPIRIT Integrated Suicide Prevention Programme: study protocol for a cluster-randomised controlled trial in rural Gujarat, India

**DOI:** 10.1186/s13063-020-04472-2

**Published:** 2020-06-26

**Authors:** Soumitra Pathare, Laura Shields-Zeeman, Lakshmi Vijayakumar, Deepa Pandit, Renuka Nardodkar, Susmita Chatterjee, Jasmine Kalha, Sadhvi Krishnamoorthy, Nikhil Jain, Arjun Kapoor, Mohammad Shahjahan, Ajay Chauhan, Filip Smit

**Affiliations:** 1 Centre for Mental Health Law and Policy, Indian Law Society, Law College Road, Pune, 411004 India; 2 Netherlands Institute for Mental health and Addiction (Trimbos Institute), Da Costakade 45, Utrecht, 3521 VT the Netherlands; 3 SNEHA Suicide Prevention Centre, Chennai, India; 4 George Institute for Global Health Elegance Tower, 311-312, Third Floor, JasolaVihar, New Delhi, 110025 India; 5 Bangladesh Centre for Communication Programs, Dhaka, Bangladesh; 6 Hospital for Mental Health, Ahmedabad, India; 7 University Medical Centers Amsterdam, location Vumc, Amsterdam, the Netherlands

**Keywords:** Suicide prevention, Child and adolescent mental health, Pesticides, Community Health Workers, mhGAP, Implementation research

## Abstract

**Background:**

Suicide is a major public health challenge globally and specifically in India where 36.6% and 24.3% of all suicides worldwide occur in women and men, respectively. The United Nations Sustainable Development Goals uses suicide rate as one of two indicators for Target 3.4, aimed at reducing these deaths by one third by 2030. India has no examples of large-scale implementation of evidence-based interventions to prevent suicide; however, there is a sizeable evidence base to draw on for suicide prevention strategies that have been piloted in India or proven to be effective regionally or internationally.

**Method:**

The SPIRIT study is designed as a cluster-randomized superiority trial and uses mixed methods to evaluate the implementation, effectiveness and costs of an integrated suicide prevention programme consisting of three integrated interventions including (1) a secondary-school-based intervention to reduce suicidal ideation among adolescents, (2) a community storage facility intervention to reduce access to pesticides and (3) training for community health workers in recognition, management, and appropriate referral of people identified with high suicidal risk.

**Discussion:**

Combining three evidence-based interventions that tackle suicide among high-risk groups may generate a synergistic impact in reducing suicides at the community level in rural areas in India. Examination of implementation processes throughout the trial will also help to prepare a roadmap for policymakers and researchers looking to implement suicide prevention interventions in other countries and at scale.

**Trial registration:**

Clinical Trial Registry of Indian Council of Medical Research, India: CTRI/2017/04/008313. Registered on 7 April 2017.

http://ctri.nic.in/Clinicaltrials/pmaindet2.php?trialid=18256&EncHid=&userName=SPIRIT

Trial registry was last modified on 28 June 2019.

## Background

Suicide remains a major public health challenge globally [1], particularly in India [2], where its complex multi-factorial aetiology permeates both health and social sectors [3–7]. In 2016, India accounted for 36.6% of all suicides in women and 24.3% of suicides in men worldwide [2]. Estimates of the total number of suicides in India vary, ranging from 230,000 to 250,000 people per year [2, 8, 9]. Suicide rates are highest amongst young people between the ages of 15 and 39 years [6, 7], and suicide has overtaken maternal mortality as the primary cause of death amongst young women in India and ranks first and second amongst the causes of mortality in women and men, respectively [2, 8]. In high-income countries, the male-to-female (M:F) sex ratio for suicide varies from 4:1 to 2:1; comparatively, in India the ratio is roughly 1.5:1 [5, 6]. There is a concomitant mental health diagnosis such as depression [5] in 90% of suicides in high-income countries; in contrast, in South Asia, this is only identified in 60% of suicides [8, 9].

An important feature of suicides largely unique to South Asia is the preponderance of pesticide poisoning as a means of suicide, particularly in rural areas. In 2015, 27.9% of suicides in India were due to poisoning, predominantly using pesticides [10]. Reducing access to pesticides in India therefore has the potential to reduce suicides in agrarian areas with access to pesticides [9]. Prior research has purported that a feasible suicide prevention strategy in India would be to study what suicide means in the local context [7, 11], reduce access to organophosphate pesticides, provide training to educators, youth, and health workers on how to provide help in times of crisis, and provide public education to improve acceptance of restricting access to means [2, 8, 11].

Global attention towards suicide prevention is articulated in the UN Sustainable Development Goals, with suicide rates as one of the two indicators for Target 3.4 (reduce premature mortality attributed to non-communicable diseases), aiming to reduce these deaths by one third by 2030 [12]. To achieve this target, evidence-based interventions need to be scaled up to result in a systemic change at national level in reducing suicidal ideation, suicide attempts and ultimately suicide rates. India has limited evidence of large-scale implementation of evidence-based interventions to prevent suicide; however, there is a sizeable evidence base to draw on for suicide prevention strategies that have been piloted in India, or proven to be effective regionally or internationally [9]. A standalone intervention is unlikely to achieve a reduction in suicide rates [13, 14]; the SPIRIT trial has therefore employed a comprehensive suicide preventive intervention programme with three evidence-based interventions. The three interventions consist of (1) a high-school-based intervention to reduce suicidal ideation among adolescents, (2) a community storage-facility intervention to reduce access to pesticides and (3) training of community health workers in early recognition, referral and the management of people at risk of suicide and self-harm.

This study is a part of the larger SPIRIT hub, which is a U.S. National Institute of Mental Health-funded collaborative hub for implementation research and a capacity-building component for a new generation of implementation researchers, to stimulate evidence-informed policy making, and influence public perceptions of suicide and attempted suicide in South Asia. Details of the SPIRIT collaborative hub are described elsewhere [Shields-Zeeman L, Vijaykumar L, Hipple-Walters B, Pandit D, Kalha J, Wensing M, et al. Building mental health and suicide prevention research and practice capacity in South Asia: an overview of the SPIRIT hub (under review)].

The SPIRIT trial will be conducted in Mehsana district, located in the state of Gujarat, Western India. Mehsana is primarily a rural district (75%), with a rural population of 1.52 million people, of which 1 million are older than 18 years [15]. Mehsana district in Gujarat was chosen as the trial site for several reasons. First, Mehsana’s rural population is engaged in cash-crop production (tobacco, pulses, castor, cotton etc.) that requires significant use of pesticides [16] and Gujarat is ranked third in the country for poisoning as a proportion of suicide deaths, and Mehsana district ranks fourth highest with respect to the number of suicides in Gujarat [17] making it one of the government’s priority districts for this trial. Second, Gujarat is one of two states in India with a state mental health policy, which emphasises the importance of addressing suicide among vulnerable groups, particularly women and farmers. Third, the Department of Health & Family Welfare, Government of Gujarat has an ongoing collaboration with the authors and is willing to partner in this trial. Fourth, Mehsana district has good availability and access to public mental health services, and is in close proximity to the State capital, where more specialised mental health services are available.

Therefore, the SPIRIT study objective is to evaluate the public health impact of an integrated suicide prevention programme implemented in 62 villages and compared to 62 control villages in Mehsana district of Gujarat, India, by evaluating:

1. The reach and adoption of the SPIRIT interventions in the target villages;
2. The effectiveness of the prevention programme in reducing suicide rates and suicidal behaviours in target populations;
3. The economic costs of delivering the suicide prevention programme

## Methods

### Design

We are conducting a cluster-randomised superiority trial with villages as clusters to evaluate the effectiveness of the prevention programme, which consists of three interventions for reducing suicides and attempted suicides. Follow-up is planned at 3 and 12 months post-baseline. We will conduct an economic evaluation alongside the trial to assess the programme costs, and a process evaluation to assess the possibility of implementing the SPIRIT programme at scale. Qualitative methods such as focus group discussions (FGDs), semi-structured interviews and document analysis will be used for the process evaluation to understand the implementation process as well as the feasibility and sustainability of the intervention.

#### Randomisation and treatment allocation

**Fig. 1:**
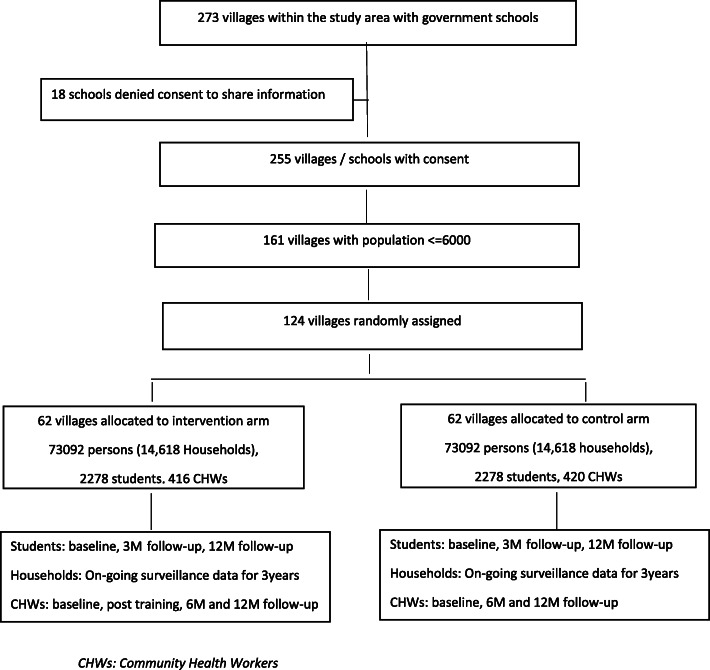
Consolidated Standards of Reporting Trials (CONSORT) flow chart. CHW, community health worker; M, month

The units of randomisation in this cluster-randomised trial are 124 villages in the Mehsana district, which an independent statistician will allocate by pre-randomisation in a 1:1 allocation ratio, to either the intervention or control arm (Fig. 1). In contrast to conventional randomisation, pre-randomisation requires that agreements of the Village Councils (Panchayats) to participate in the project must be obtained after (not before) randomisation. This procedure allows us to have a higher accrual rate than under conventional randomisation. We also anticipate that pre-randomisation will address the problem of contamination in our study. We are concerned that if Village Councils were consented to participate in the trial prior to randomisation, some Village Councils may on their own accord decide to implement the intervention if they were subsequently allocated to the control arm of the trial. On the other hand, in our chosen procedure, Village Councils will have a choice either to participate in the study - in the pre-randomised arm - or not participate at all. At this stage, we do not foresee a problem of loss of autonomy of the village as a participating unit given that there are no other suicide prevention programmes simultaneously running in the district. To further minimise the risk of contamination, intervention and control villages will be geographically separated by a minimum of 5–7 km and villages within 5 km of each other or villages where children from two or more villages attend the same school in one village will be considered as a single unit prior to randomisation. We also have obtained approval of the Ethics Committee for pre-randomisation.

### Conditions

#### Intervention condition

The integrated suicide prevention programme consists of three interventions, implemented in intervention villages.

##### Intervention 1: school-based intervention (Youth Aware of Mental health (YAM))

The school-based intervention consists of a universal mental-health promotion programme in schools within intervention villages aimed at preventing depression, reducing suicidal ideation, and promoting mental health among students in grade 9 who are between 14 and 16 years of age. We chose this age group as suicide rates begin to increase from age 15 years onwards in South Asia [6]. In addition, psychopathological and behavioural changes often have their onset in adolescence, making mental health prevention and promotion particularly important in this target group [18, 19].

The school-based suicide prevention programme consists of a locally adapted version of the Youth Aware of Mental Health Programme (YAM), which has been effective in reducing suicide attempts and severe suicidal ideation among 14–15-year-old adolescents in a European multi-centre, multi-country, randomised controlled clinical trial [18]. YAM is an interactive programme for adolescents delivered within a teacher-free space, aiming to promote discussion and increase knowledge about mental health and the development of problem-solving skills and emotional intelligence. YAM is designed to help improve coping skills to deal with stressful life events that can trigger suicidal behaviours. YAM is a manualized 5-h programme broken down into 3-h of roleplay sessions and 2-h of interactive lectures and discussions about mental health at the beginning and end of the intervention. In addition, students receive a booklet on mental health issues and strategies to deal with difficult life events. Educational posters are available for each classroom; the themes of the posters are related to mental health and suicide, adapted by the SPIRIT research team to the local language and to the local setting in Gujarat [19].

Extensive adaptation of the intervention was undertaken to make the YAM materials relevant to the Indian context. Materials (booklet, posters, opening session slides and instructor manual) were first reviewed by the project team to assess where original materials (and language used) were feasible for use in the Indian context, noting any challenging language or concepts in an implementation log. Materials were then adjusted accordingly and reviewed by the original developers of the intervention. The adapted materials were translated into local language (Gujarati) and piloted with youth in schools. Focus group discussions on the programme with the youth and adult stakeholders from the local education, health and social services departments formed an essential part of the adaptation process. Recommendations from the FDGs and the pilot were used to further tailor the materials for the local context. Final versions of the materials were translated and back-translated, and further reviewed by a native speaker for language inconsistencies. The materials were then sent for creative design to produce the final Gujarati adapted YAM programme.

##### Intervention 2: community storage of pesticides

Suicide through pesticide ingestion accounts for a large proportion (27.9%) of suicides in India [9], and thus a key suicide prevention intervention is reducing access to means of suicide; one such strategy is through safe storage of pesticides in communities [8]. Reducing access to the means of suicide in South Asia may be particularly effective given that most suicides are impulsive and not related to a concomitant diagnosis of mental illness [5]. Previous studies in South Asia have explored the feasibility and the implementation of community storage facilities for pesticides, a community-based solution to reducing access to means of suicide [20, 21]. The strategy has two distinct advantages [20]: (1) it reduces the storage of pesticides in homes and (2) it engages and involves the entire community, including the Village Councils, in the prevention of suicide.

This intervention consists of setting-up community storage boxes placed in the Village Council office premises or at a central location. Each farming household is offered a locker at this facility free of charge and the family is encouraged to store all pesticides in this box. A trained attendant (facility manager) will be stationed at the community storage facility and will document use of the boxes on a day-to-day basis.

##### Intervention 3: community health worker (CHW) training in identification, support and referral of persons with suicidal risk and behaviour

This intervention consists of training and a follow-up programme for CHWs in the intervention villages to identify people at risk of self-harm and suicide and to support and refer such persons to appropriate local services. The training is based on the World Health Organization Mental Health Action Programme (mhGAP), Self-harm/Suicide module [22, 23] initially designed to be used by medical practitioners in low-income and middle-income countries. This mhGAP module has been adapted by the SPIRIT research team with feedback from 10 national and international experts for use by non-specialised CHWs in rural settings through a structured consultation process. The adapted module resulting from these consultations was piloted through two FDGs among a selection of healthcare professionals and community health workers (*n* = 10) in Mehsana district for feasibility and appropriateness. The final module was translated into Gujarati and piloted again with CHWs in the district from villages that are not part of the SPIRIT trial. Any further changes were then integrated into the module to arrive at the final version in Gujarati. The training module includes information on how to identify and act in cases of persons at risk of self-harm and suicide, when and to whom to refer, and type of psychosocial support to provide at different stages.

#### Comparison condition

Residents in the control villages will not receive any intervention to prevent suicide other than enhanced usual care, which involves provision of brochures with information on available mental health services and other resources for seeking help, such as emergency helpline numbers and contact details of public and non-governmental healthcare services.

### Sampling units

#### Primary sampling units: villages

A village is regarded as a primary sampling unit (cluster) and qualifies for study inclusion if:

1. The Village Council has agreed to participate in the study
2. There is a high school in the village with more than 35 students and the school has agreed to participate in the study
3. The total population of the village is ≤ 6000 people (the population size was determined considering the distance travelled by villagers to access the central storage facility (CSF))
4. The primary occupation of the villagers is farming or agriculture-related work involving use of pesticides

#### Secondary sampling units: participants

The study consists of adolescents in grade 9 in public high schools (14–16 years of age) and adult community members (18 years and older) residing in rural villages in Mehsana district.

#### Sample size and power calculation

The targeted effect of the SPIRIT intervention is to reduce suicide and attempted suicides by 20%. This target was validated through a series of interviews we carried out among Indian psychiatrists to build consensus on whether this would constitute a clinically meaningful outcome. This outcome will be measured using data on suicides and attempted suicides gathered through the surveillance system as described below. The sample size for each intervention was calculated separately in this cluster-randomised controlled trial.

#### Sample size for intervention 1 (school-based prevention)

The prevalence of suicidal ideation among adolescents in India has been estimated at 19% [24]. Given power of 80% and alpha of 0.05 (two-tailed), the required sample size to detect a 20% reduction in suicidal ideation from 19% to 15% is 2866. Adding a design effect of 1.59, the sample is 4557 (*n* = 2278 students each across the schools in the intervention villages and schools in the control villages).

#### Sample size for intervention 2 (community storage facilities for pesticides)

SPIRIT aims to reduce the suicide and attempted suicide rate by 20% through the community storage facility for pesticides. India’s National Crime Records Bureau (NCRB) reported the Gujarat state-level suicide rate for 2014 and 2015 as 11.8/100,000 persons. While some studies from rural southern India using verbal autopsies on suicide victims have reported annual suicide rates as 62/100,000, and rates of 148/100,000 and 58/100,000 in young men and women (10–19 years of age), respectively [24, 25], there are no national prevalence data for attempted suicides in the country or for Gujarat state. Indian and European studies have estimated that suicide attempts are usually anywhere from 10 to 40 times more frequent than completed suicides [26, 27]. We therefore estimated the rate of suicides and attempted suicides as 10 per 1000 population as estimated recently by other studies in India [20].

To detect an absolute difference of at least 20% in the prevalence of suicide and attempted suicide after the intervention, a total sample size of 60,901 is needed, calculated at a significance level of 0.05 with power of 80%. As the cluster (village) sizes are unequal, the variance inflation factor (VIF)/design effect is applied to achieve the effective sample size. This is a function of the average cluster size, *m*, variation in cluster size, cv^2^, and the intra-cluster correlation (rho) (VIF = (1+ ((cv^2^ + 1) m-1) rho). Compensating for clustering with the design effect, 121,820 persons are required. After considering 20% attrition, a population of 146,184 individuals is required for the study - 73,092 persons in the control and 73,092 in the intervention arm (total of 29,236 households; 62 villages per arm; total of 124 villages). Assumptions about the number of households per village (*n* = 522) in Mehsana district and the average number of household members (*n* = 2509) are based on the Census of India (2011). Thus, 124 clusters/villages are randomised, with 62 each in the control and the intervention arm to account for village refusal rate.

#### Sample size for intervention 3 (CHW training)

**Fig. 2:**
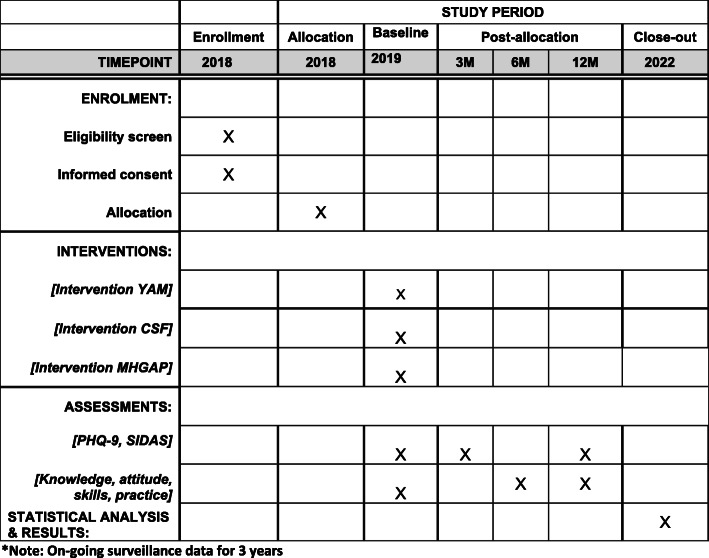
Time schedule of enrolment, interventions and assessments. YAM, Youth Aware of Mental Health; CSF, central storage facility; mhGAP, Mental health Gap Action Programme; PHQ-9, Patient Health Questionnaire-9; SIDAS, Suicidal Ideation Attributes Scale; M, month

There are approximately 416 CHWs based in 62 intervention villages and 420 CHWs in 62 control villages. All CHWs in the intervention and control villages will be enrolled as participants in the trial and therefore, strictly speaking, these numbers are not a sample size calculation. The schedule of enrolment, intervention delivery and impact assessment is described in Fig. 2.

#### Recruitment

All individual participants are recruited from the pre-randomised clusters based on their intervention-specific inclusion and exclusion criteria. For intervention 1, adolescents attending school in the 9th grade, who have parental consent, assent to participate and do not have a learning disability will be recruited into the trial.

For intervention 2, all agricultural households in the intervention villages will be invited and encouraged to use the community storage facilities. Each farming household will be allocated one storage box and consent will be obtained from the head of the household for collecting data related to the household’s use of the storage box.

For intervention 3 (CHW training), the decision to participate or not in the training of CHWs in the trial is not a village-level decision, but a decision that comes from the Department of Health and Family Welfare, State Government of Gujarat, who employ all health workers. The Department of Health and Family Welfare is a formal collaborator in the SPIRIT project team and CHWs are expected to attend this training. However, consent will be obtained from all the CHWs working in intervention villages (participants at the training sessions) and control villages (all those included in the trial) for collecting data for the trial. For the purposes of this study, we will include the following types of community-based health workers:

1. Primary healthcare workers working at primary care centres;
2. Community-based health workers (Anganwadi workers, Auxiliary Nurse midwives and multipurpose health workers who provide basic health care at the village level); and
3. Lay health workers (Accredited Social Health Activists, also known as ASHAs).

### Measures

The primary outcome of the integrated suicide prevention programme is a reduction in suicides and attempted suicides in the intervention villages compared to control villages. In addition, each intervention has its own primary and secondary outcomes (Table 1).

**Table 1:** Primary and secondary outcomes of specific interventions of the cluster-randomised controlled trial

Interventions	Primary outcome	Secondary outcomes
Intervention 1: suicide prevention in schools through the Youth Aware of Mental Health (YAM) intervention	Reduction in suicidal ideation at 3-month and 12-month follow-up among adolescents in intervention schools	▪ Proportion of schools that agreed to implement the YAM intervention of the total number of schools approached for implementation ▪ Of those schools that agreed to implement the YAM intervention, the proportion of schools that implemented the programme ▪ Proportion of adolescents in intervention schools who received the YAM programme out of total number of adolescents in the selected grade
Intervention 2: community storage facilities (CSF) for pesticides	Reduction in the number of suicides and attempted suicides in intervention villages at 12-month follow-up	▪ Proportion of villages of those that were approached that agreed to have the CSF ▪ Proportion of villages that agreed to have the CSF or provided for or arranged space to build the storage facility ▪ Proportion of households reached with promotion activities linked to community storage of pesticides; proportion of households that requested a community storage box; proportion of households that received a community storage box ▪ Proportion of households in the villages that used the boxes regularly
Intervention 3: community health worker (CHW) training for identification, support and management of people at risk of suicide or self-harm	Number of persons with suicidal ideation detected by trained CHWs and number of persons referred by CHWs to mental health professionals for care for suicidal ideation	▪ Proportion of health workers trained in intervention villages ▪ Improvement in knowledge, skills, attitudes and practice of CHW who received training ▪ Number of CHWs applying knowledge and skills obtained through the training programme in their day-to-day practice at 6-month and 12-month follow-up

#### Primary outcome of the integrated suicide prevention programme

Suicide data in India are officially and publicly reported on an annual basis by the NCRB. The Bureau collates suicide data from police records across the country but does not record attempted suicides. In 2015, NCRB estimated India’s suicide rate at approximately 10.6 per 100,000 [10] compared to the WHO estimate of 15.7 per 100,000 for the same year [9] suggesting that NCRB data may be a significant underestimate of the true suicide rate. The Million Death Study [6] also concluded that NCRB underestimates suicide deaths in men by at least by 25% and in women by at least 36%, given that three fifths of total suicides occur at home and are unreported. Prior research has shown that underreporting happens due to classifying deaths due to suicide under various “hidden” causes [11, 28] such as accidental deaths, illnesses and/or undetermined deaths, drug poisoning and intoxication [11], fear of police investigations [27] and stigma associated with suicides and attempted suicides. Data on attempted suicides, “simple hurts” are clustered together under the umbrella of “Other IPC Crimes” [29]. There are no data on attempted suicides given in the official records in India.

To obtain a reliable estimate of suicide deaths and suicide attempts in Gujarat, we designed and implemented a surveillance system to collect data on suicides and attempted suicides from community-based key informants. The surveillance system has been described extensively elsewhere [Vijayakumar L, Pathare S, Jain N, Nardodkar R, Pandit D, Krishnamoorthy S, et al. Implementation of a community-based suicide surveillance system in rural India, (submitted)]. Briefly, the SPIRIT surveillance system is based on methodology used to establish surveillance systems for disease monitoring in other countries [30, 31], adapted for the Indian context and for collection of data on suicidal behaviour.

The surveillance system collects information from third-party key informants within the community. Key informants are chosen based on their work profile, knowledge and understanding of the village geography and socio-cultural dynamics. Key informants are representative of the village population and represent diverse communities covering all areas within the village.

Informants are approached every month by trained staff to gather details about individuals in their village who have either died due to suicide or have attempted suicide. Details are recorded in a confidential case report form (CRF), due to the sensitive nature of the information. Data on attempted suicides and suicides are also collected from public and private health facilities in the district and police records. Data received from multiple key informants, health facilities and police records are collated, cross-verified and triangulated by trained independent research staff (not research staff who collect the data). This process helps in identifying unique individuals who have died by suicide or attempted suicide and ensures that individual instances of suicide or attempted suicide are not counted more than once. These details are then de-identified and recorded in a database.

#### Intervention 1: YAM in schools: primary outcome

Suicidal ideation among young adolescents in both intervention and control condition schools will be assessed using the Suicidal Ideation Attributes Scale (SIDAS) [32]. SIDAS is a 5-item self-report scale addressing attributes of suicidal thoughts: frequency, controllability, closeness to attempt, level of distress associated with thoughts and impact on daily functioning. Scores ≥ 21 indicate a high risk of suicidal behaviour among youths. In this study, the SIDAS score will be assessed at baseline and at 3-month and 12-month follow-up. If a student has elevated scores on suicidal ideation (≥ 21 out of a maximum score of 50) at baseline assessment, he/she will be referred to the closest locally available health services such as a primary care centre, district mental health hospital or the Hospital for Mental Health in Ahmadabad (tertiary specialised mental health hospital).

Depression symptoms will be assessed using the Patient Health Questionnaire-9 (PHQ-9), a 9-item self-report scale [33, 34]. The PHQ-9 will be administered to students in the intervention and control schools at baseline and 3-month and 12-month follow-up. PHQ-9 scores range from 0 to 27 with higher scores indicating greater symptom severity. Scores of 5, 10, 15 and 20 represent cut-offs for mild, moderate, moderately severe and severe depression, respectively. Students who meet criteria for severe depression (scores > 20 on the PHQ-9 scale) at baseline assessment will be referred to the closest locally available health services such as a primary health centre, district mental health hospital or the Hospital for Mental Health in Ahmedabad (tertiary specialised mental health hospital).

##### Secondary outcomes

Data for secondary outcomes (Table 1) will be collected from process measures maintained by the SPIRIT research team, such as a log with all the schools approached for the intervention and a record of all the schools that agreed to implement the intervention (see Table 1 for further description of process measures).

##### Process measures

Focus group discussions will be conducted with YAM instructors to obtain more in-depth information on the acceptability, feasibility, and perceived impact of the delivery of YAM sessions in intervention schools.

#### Intervention 2 community storage facility for pesticides: primary outcome

The primary outcome for intervention 2 is the same as the primary outcome for the integrated suicide prevention programme - namely reduction in deaths due to suicides and attempted suicides, which will be measured by the surveillance system as described above.

##### Secondary outcomes

Data for secondary outcomes (Table 1) will be collected from data logs maintained by the SPIRIT research team, such as a log of all the villages approached for installation of storage boxes, number of villages that agreed to have the storage boxes, number of households that requested a storage box and number of households that were allocated a storage box.

Monthly log records of the CSF for pesticides (maintained by the facility manager) in the intervention villages will be analysed to evaluate its use. Additionally, survey responses from a structured questionnaire administered by trained data collectors among households in four villages (two control villages, two intervention villages) will be assessed to gain an understanding of current purchase, use and storage options for these pesticides. Additional information will be collected at follow-up about the acceptability and use of the CSF in the intervention villages.

##### Process measures

In-depth interviews and FGDs will be conducted with facility managers, relevant household members, and village heads to understand reasons for use and/or non-use of the community storage facility for pesticides in the intervention villages. A random sample from users (*n* = 5) and non-users (*n* = 5) of the storage facility will be interviewed in depth to identify implementation barriers and facilitators related to use of the community storage facilities.

#### Intervention 3: primary outcome

The primary outcome measure for intervention 3 is change in knowledge, attitude, skills and practice (KAP) among community health workers. Assessments of KAP will be conducted at baseline (pre-training) and post training in the intervention villages. In both intervention and control villages, KAP assessments among CHWs will be carried out at 6-month and 12-month follow-up.

Knowledge will be assessed using a self-report gatekeeper training questionnaire derived from previously validated questionnaires in gatekeeper training surveys [35], with one item adapted to Indian settings. The questionnaire consists of eight questions gauging knowledge about suicides and suicidal behaviour; talking to someone who is suicidal and arranging help; and ability to manage clients with a high risk of suicide in a timely and appropriate manner.

Skills will be assessed using eight multiple-choice questions adapted from the WHO mhGAP self-harm/suicide module [13]. Items focus on what to do when someone presents with ideas of self-harm/suicide, understanding co-morbidity as a risk factor, asking someone directly about self-harm, emergency management, best interventions, talking to a care-giver and other psychosocial interventions for self-harm/suicide.

Attitudes among the CHWs towards suicide prevention will be assessed using a 14-item attitude questionnaire developed initially in the USA by Herron et al. (2001) for suicide-prevention front-line health staff [36]. Items focus on how staff perceive responsibility for suicide prevention, on funding suicide prevention services, and on working with persons at risk of suicide and attempted suicide.

To measure how knowledge is translated into practice post-training, CHWs will be asked to maintain a diary recording details of identification, assessment and referral to health services of any clients who show possible suicidal ideation or risk of self-harm or suicide. After referral, each client is expected to be followed up by a CHW nine times over the course of next 12 months and notes of the client consultation will be documented by the CHW using a structured follow-up questionnaire. The diary provides a record of the number of persons a CHW has seen with suicidal ideation or risk of self-harm, and the number of people assessed by trained CHWs and referred to mental health professionals. The average time to complete the KAP questionnaire is 45 min and each follow-up questionnaire takes 20–25 min. The diary provides a record for the primary outcome of this intervention, namely the number of persons with risk of suicide identified, assessed and referred by trained CHWs to mental health professionals and followed-up by CHWs.

##### Process measures

In-depth interviews and FDGs will be conducted with CHWs in the intervention villages to explore facilitators and barriers to the implementation of the intervention and to understand the impact of the training on their daily work as a CHW. In addition, semi-structured interviews with end-users (e.g. patients) will be conducted to understand their experience of care and services provided by the CHW.

##### Intervention costing

We will analyse costs which will be confined to the costs of implementation and intervention, and the intervention impact will be defined as the reduction in the number of suicides in the experimental villages relative to the number in the control villages [37]. This will shed light on incremental costs per averted suicide. The costs will be estimated from a government provider perspective and economic costs (actual financial outlays and cost in time spent related to the intervention) will be considered [38]. We will also estimate the additional resource requirements for the government to scale the intervention.

An effective suicide prevention programme is defined as one that will lower the healthcare utilisation costs of people who attempt suicide and end up in the hospital emergency room (thus also quantifying the cost-offsets of the intervention). Cost-offsets could also occur when fewer people attempt suicide under the impact of the prevention programme, and thus continue to be productive members of society (less absenteeism from work). These cost-offsets outside the intervention will be calculated.

### Adverse events

Even though the possibilities of adverse events directly related to the intervention are low, the SPIRIT study will maintain an incident register to record serious adverse events (SAEs) and adverse events (AEs). SAEs in this study are defined as attempted suicide, completed suicide, death due to other reasons or hospitalisation for an acute psychiatric crisis among students participating in the YAM intervention. AEs include emotional distress caused by a trial procedure (either the outcome assessments or the intervention delivery). Recognition of SAEs and AEs is a standard training module for all SPIRIT research team members. Ongoing identification of SAEs is monitored by the surveillance system data collection team. In the school-based intervention, information about any SAEs may be reported by parents, data collectors, teachers and/or school heads. For the community storage boxes, SAEs may be reported by the CHW and/or the attendant of the community boxes and/or any other community member. A protocol specifying questions for each SAE/AE has been established and each project team member trained in this protocol. Additionally, a protocol for reporting AEs to the Principal Investigator (PI), Data and Safety Monitoring Board (DSMB) and Institutional Review Board (IRB) is in place for the duration of the project.

### Data collection and management

#### Data collection

Data are collected at the individual level (demographic data, data collected using standardized tools/questionnaires), household level (information on the head of the family, structured questionnaires, daily records of use of the CSF) and village (community) level. Field-based data collection will be paper-based and will be done by trained data collectors. Prior to starting data collection, all research staff will be trained in protection of human subjects, good clinical practice guidelines and administration, data entry and analysis of study-specific data collection tools. All the data collectors, trainers and research officers will receive refresher training at regular intervals to ensure data quality and adherence to intervention protocol. The YAM follow-up data collection schedule will be explained to the parents of child participants at the time of the informed consent/assent procedure. Quarterly review and facilitation meetings will be organised at the village level for retaining the participants in the CSF and mhGAP intervention. Three follow-up visits will be conducted if participants are absent for whatever reason when the data collection is due. Written Informed consent will be sought from all participants. Consent of an illiterate participant will be obtained in the presence of a literate impartial witness according to the guidelines of the Indian Council of Medical Research [39]. In intervention 1, consent of one of the parents and assent of the adolescent will be obtained. For intervention 2, consent of the head of the household (one who manages the use of pesticides at home) will be sought. Similarly, consent of the head of the household will be sought for collecting data on use of community storage boxes. For intervention 3, consent of all the CHWs will be obtained only for data collection. For surveillance data collection consent of all the key informants will be obtained. Each study participant will receive a 5-digit alphanumeric unique study ID. The collected data will be de-identified prior to entry into the electronic database.

#### Data management

SPIRIT will develop and implement a comprehensive data management system allowing for collecting high-quality data by maintaining ongoing on-site and off-site quality assurance and quality control checks. Research staff handling study data will receive extensive training in procedures for handling sensitive information, accurate data entry, surveillance and intervention-specific data storage and data archive. All completed forms will be checked for completeness and accuracy, first by data collectors and later by a research associate responsible for data management. During monthly monitoring visits, the data manager will audit hard copies of the completed CRFs to check for completeness and accuracy. Prior to entering the data into an electronic data capture interface, all study data will be anonymized. A separate record will be maintained linking identifiable information and unique study IDs. Participant-specific data will be entered into the electronic data capture interface using only study IDs. Data entered will be cross-checked and an audit trail will be maintained if corrections in the data are warranted. Raw data will not be uploaded onto the Internet; instead an electronic data capture interface will function offline, and data will be transferred from Mehsana to Pune (where the project coordination office is) from the site on a monthly basis using encrypted and protected hard drives. The data manager will maintain the database with cleaned data, which will be used by the statistician based in Pune for data analysis.

#### Data storage, security and confidentiality

All consent and assent forms and records of personally identifiable information will be kept confidential and protected from unauthorized access. Hard copies of the CRFs obtained from the surveillance system will be stored by month of surveillance, whereas hard copies of the interventions-specific data will be stored by village where the data were collected. All data will be kept in locked cupboards, accessible by key only by the data management staff and the principal investigators only.

#### Data monitoring

The trial is monitored by an independent DSMB. The DSMB is made up of five members and consists of independent experts in biostatistics, health services research, medical ethics, public health, social science research and clinical trials within the Indian context; it will meet twice a year to oversee the trial. The trial is also subject to quarterly independent quality assurance audit of the performance of the site as per the guidelines of the funding agency.

### Data analysis and reporting

All findings from the trial will be reported in accordance with SPIRIT, Extended CONSORT and CHEERS guidelines. For analysis of quantitative data, we will first tabulate descriptive statistics for all study variables to understand trends in the data. For efficacy analysis of the primary outcome, we will apply an intention-to-treat (ITT) approach to all participants assigned to the intervention arm as randomised, regardless of the study intervention received. We will test the null hypothesis of no difference in the population incidence of suicides and attempted suicides between clusters allocated to the intervention and control arms, using surveillance data.

A multilevel Poisson regression model will be employed, with inflated standard error to accommodate the clustered design. Inflation will be achieved using a normal or gamma distributed random effect, whichever best describes the variation in number of suicides across villages. Change in suicide and attempted suicide rates from baseline to follow-up (12 months) at intervention and control sites will be calculated separately, adjusting for random cluster effect. We will compare this change in the intervention and control arms and calculate the 95% confidence interval.

If there is statistical evidence of over-dispersion in the Poisson regression model, a negative binomial regression analysis will be performed. Allowance will be made if a greater than expected number of clusters without a case of suicide/suicide attempt is observed, using the Stata zero-inflated negative binomial regression (“zinb”) command or zero truncated Poisson regression for instance, which includes a test of whether this approach offers an improvement over the standard model. All hypothesis testing will be carried out at the 5% (two-sided) significance level. *P* values will be rounded to 2 decimal places. *P* values below 0.05 will be reported as < 0.05 in tables.

#### Specific analysis for each intervention

For intervention 1, differences in PHQ-9 and SIDAS scores at 12-month follow-up between students in the intervention and control arms will be calculated using a multilevel, linear effect model adjusting for clustering by villages as a random effect. We will analyse the SIDAS scores both on a continuous and categorical scale. Parameters for categorical variables will be either no ideation versus ideation or tertile scores (low/medium/high).

For intervention 2, all the secondary outcome data as per Table 1 will be analysed and presented as summary tables using count data and logs maintained by research team. Additionally, data from survey responses from a structured questionnaire from four villages (two control, two intervention) will be analysed pre and post intervention to evaluate pesticide purchase, storage options and acceptability and use of the CSF in the intervention villages.

For intervention 3, due to the ordinal nature of the data (knowledge, skills and attitude scales), multilevel ordered logistic regression (ordinal logistic regression) will be employed to assess change in knowledge, skills and attitude from baseline to follow-up in both intervention and control groups. Adjusted and unadjusted estimates will be reported as the proportional odds ratio or log odds coefficient. The number of persons with suicidal ideation detected and referred on by trained CHWs will be recorded as count data through logs and daily record registers. All the count data will be analysed using descriptive statistics.

#### Secondary outcomes

Secondary outcome data will be obtained from routinely collected information through the count data (programme data) from records, registers and review documents on response rate only in the intervention villages. It will be complemented by qualitative data analysis.

#### Missing data

Participants in either study arm who have incomplete data/no data by the end of the study will be considered as missing data/lost to follow-up. No imputation will be made for participants with missing data. Subgroup analyses may be performed for participants with full versus partial intervention if appropriate. Similarly, we may perform sensitivity analysis to explore the impact of non-informative loss to follow-up by conducting a complete case analysis.

#### Qualitative process evaluation data

Qualitative interviews will be recorded and analysed thematically, using both inductive and deductive coding approaches. Two coders will independently code each transcript by identifying emergent themes and also will code themes from the interview guides. Broader themes will be broken down into smaller, more specific sub-themes until no further subcategories are required. Inter-coder reliability will be assessed; agreement between 66 and 97% is taken to indicate good reliability. Any coding disagreements will be resolved through project team discussions and where required, a third independent coder will re-code the transcript. Audit trails of ongoing decisions including revision of existing codes and emergent codes will be kept for all interviews.

## Discussion

This study aims to explore and assess the public health impact and translation into practice of an integrated suicide prevention intervention by evaluating the following elements: (1) effectiveness of the intervention in reducing suicide rates and suicidal behaviours in the target population in a district in rural Gujarat; (2) adoption of the intervention by villages, schools and primary healthcare system in a district in rural Gujarat and (3) cost of the delivery of a complex suicide prevention programme in a district in rural Gujarat. This trial will contribute to increasing our knowledge and understanding of key components for scaling up the suicide prevention intervention in Gujarat and with the ultimate aim to narrow the implementation gap in Gujarat and South Asia and other similar contexts.

There are significant expected implementation challenges to note. First, the trial involves multiple sectors including health, education and agriculture at the state government and local village level and building an effective partnership is critical to the success of this suicide prevention programme. Second, translating and adapting the YAM intervention and mhGAP interventions for use in India required an extensive adaptation period, involving deconstructing the YAM and mhGAP training content and manual. A particular focus of adaptation was incorporating the instructors’ experiences in the field, considerations of socio-cultural factors and an extensive translation and back-translation process, followed by pilot testing the manuals for feasibility and acceptability.

## Limitations

All interventions, the surveillance system, and scales used to measure different outcomes are extensively adapted and contextualized for the target population. Scaling up to other contexts may be challenging due to the extensive adaptation process required if implemented in other low-income and middle-income country contexts, thus affecting generalisability of the intervention.

Cross-contamination across two arms (under intervention 3) can be expected in rare cases of transfer of participants (CHWs are public servants and are occasionally transferred from one village to another village) from an intervention to a control village during the study period, which may have some effect on the study findings.

Information collected from adolescents (under intervention 1) through the SIDAS and PHQ-9 is self-reported and may be subject to self-report bias. The primary outcome measure is based on data collected through the surveillance system and hence the quality of these data will significantly impact the results. This has been addressed by extensive training and re-training of the data collection staff and triangulation of the data by independent research staff to ensure quality of the data collected.

## Conclusion

If successful, the integrated intervention has the potential to constitute an integrated evidence-based practice for suicide prevention in India, and potentially other low-income and middle-income country contexts. As the intervention combines three evidence-based interventions that tackle suicide among high-risk groups (adolescents and young people, rural community members), this may allow for more synergistic impact in reducing suicides at the community level. Examination of implementation processes throughout the trial will also help to prepare a roadmap for policymakers and researchers looking to implement suicide prevention interventions in other settings, or at scale.

## Trial status

Protocol version: SPIRIT Data and Safety Monitoring Plan V 1.7 August 2018

Protocol modification in Clinical Trial Registry - CTRI trial registry was last modified on 28 June 2019.

Date of start of recruitment - July 2019

Date of recruitment completion - September 2020 (recruitment of participants is currently ongoing. CTRI registry lists recruitment as open)

## Data Availability

The datasets will be available to appropriate academic parties on request from the principal investigator, in accordance with the data sharing policies of the institute and funding agency within one year of completion of complete analysis of the data.

## Abbreviations

AE: Adverse events
CHW: Community health worker
C-RCT: Cluster randomised controlled trial
CRF: Case report form
CSF: Central storage facility
DSMB: Data and Safety Monitoring Board
FGD: Focus group discussion
ICC: Intra cluster correlation
IRB: Institutional Review Board
ITT: Intention to treat
mhGAP: Mental health Gap Action Programme
NCRB: National Crime Records Bureau
NIMH: National Institute of Mental Health
PHQ-9: Patient Health Questionnaire-9
PI: Principal Investigator
SAE: Serious adverse event
SIDAS: Suicidal Ideation Attributes Scale
TOT: Training of the trainers
YAM: Youth Aware of Mental Health
WHO: World Health Organization
